# Automated Segmentation of the Deep Inferior Epigastric Artery on CTA Using Deep Learning for Preoperative Planning of DIEP Flap Breast Reconstruction

**DOI:** 10.3390/s26216771

**Published:** 2026-10-26

**Authors:** Cong Su, Zitan Shi, Pengfei Wei, Weiqing Zhao, Shanshan He, Shu Wang, Jian Yin

**Affiliations:** 1 The Department of Breast Reconstruction and Oncoplastic Surgery, Tianjin Medical University Cancer Institute & Hospital, National Clinical Research Center for Cancer, Key Laboratory of Breast Cancer Prevention and Therapy, Tianjin Medical University, Ministry of Education, Key Laboratory of Cancer Prevention and Therapy, Tianjin, Tianjin’s Clinical Research Center for Cancer, The Sino-Russian Joint Research Center for Oncoplastic Breast Surgery, Tianjin 300060, China; sucong5858@163.com (C.S.); zhaowq1123@163.com (W.Z.); ssh85@yahoo.com (S.H.); ekinmagica@163.com (S.W.); 2 School of Mechanical Engineering, Tianjin University, Tianjin 300350, China; zitan@tju.edu.cn (Z.S.); 2024244251@tju.edu.cn (P.W.); 3 Institute of Medical Robotics and Intelligent Systems, Tianjin 300072, China

**Keywords:** breast reconstruction, deep inferior epigastric artery, deep learning, image segmentation

## Abstract

Accurate segmentation of the deep inferior epigastric artery (DIEA) on computed tomography angiography (CTA) is important for preoperative planning of deep inferior epigastric perforator (DIEP) flap breast reconstruction. This retrospective study established a dedicated DIEA dataset and developed a deep learning model for automated segmentation. A total of 4446 high-resolution abdominal CTA images from 31 patients were curated and annotated at the pixel level using a multi-reviewer protocol. An improved ResU-Net++ model was trained with Tversky loss, AdamW optimization, and cosine annealing, and was compared with U-Net, ResU-Net, and ResU-Net++ using Accuracy, Sensitivity, Intersection over Union, Dice Similarity Coefficient (DSC), and Hausdorff Distance (HD). The proposed model achieved a Dice Similarity Coefficient (DSC) of 0.6807, an IoU of 0.5556, and a Sensitivity of 0.7326, representing the highest values for these metrics among the evaluated models. The complete automated processing pipeline required 0.0093±0.0015 s per image, compared with 19.85±5.75 s for manual segmentation (p<0.001). These findings support the technical feasibility of automated DIEA segmentation on abdominal CTA. Its potential application in DIEP flap preoperative planning and clinical workflows requires further prospective validation.

## 1. Introduction

Breast cancer remains a leading oncological concern, with surgical management increasingly prioritizing oncological safety alongside optimal aesthetic and functional reconstruction [1,2,3,4,5]. Autologous tissue breast reconstruction, particularly the deep inferior epigastric perforator (DIEP) flap, offers the advantage of a more natural symmetry and texture, leading to greater patient satisfaction, enhanced psychosocial well-being, and improved sexual healt [6,7,8]. Computed tomography angiography (CTA) is the gold standard imaging modality for preoperative assessment of the vascular anatomy for DIEP breast reconstruction [9,10,11]. In the conventional protocol, the completion of an abdominal CTA scan necessitates that surgeons or radiologists employ various post-processing applications to construct 3D models of the deep inferior epigastric vessels and abdominal wall. This traditional image analysis is heavily reliant on the manual segmentation and labeling of the deep inferior epigastric artery (DIEA) by radiologists. This labor-intensive and subjective process is not only inefficient but also introduces significant potential for error and inconsistency. The challenge is magnified when processing large-scale medical imaging data, making manual segmentation highly impractical, which in turn escalates the workload and diminishes both the reproducibility and precision of the outcomes. Thus, an automated segmentation method for the DIEA is exceptionally crucial.

However, this task faces numerous challenges for several reasons. First, the anatomy of the DIEA—such as its origin, course, and branching patterns—can exhibit significant inter-individual variability [12,13]. Second, some CTA images may suffer from artifacts, noise, or poor clarity. These confounding factors can obscure the vascular structures, making them more difficult to identify and thereby compromising the final segmentation quality. Additionally, the DIEA is typically a thin vessel with intensity and texture characteristics similar to those of surrounding tissues. This often results in faint and poorly defined boundaries on imaging. Unlike other established medical imaging datasets for structures, annotated imaging data for the DIEA remains relatively scarce [14,15,16]. This scarcity hinders the development and robust validation of deep learning-based automated segmentation algorithms.

To address this unmet need, this paper introduces a dedicated DIEA dataset, curated from 4,446 high-resolution patient abdominal CTA scans, each with pixel-level manual annotations performed by medical experts. We propose a novel vascular segmentation network for this vasculature and provide a comprehensive assessment of its efficacy.

## 2. Materials and Methods

This retrospective study was approved by the Medical Ethics Committee of Tianjin Cancer Hospital; as it involved a retrospective analysis of existing imaging data, the requirement for written informed consent was waived.

### 2.1. Data Acquisition

For model development and validation, abdominal CTA images were retrospectively collected from consecutive patients who underwent DIEP flap breast reconstruction at the Department of Breast Reconstruction and Oncoplastic Surgery, Tianjin Medical University Cancer Institute and Hospital, between January 2011 and July 2021. Inclusion criteria were: (a) scheduled for DIEP flap breast reconstruction in our department with a preoperative abdominal CTA examination and (b) availability of complete CTA data with high image quality and clarity. Exclusion criteria were: (a) partial or complete loss of CTA image data or (b) suboptimal vascular opacification or any other issues that precluded reliable vascular segmentation.

Using 64-slice IQon Spectral CT scanner (Philips Medical Systems Nederland B.V., Best, The Netherlands) with contrast tracking trigger technology in the arterial phase. The tracking position was set in the external iliac artery, with the trigger threshold of 100 HU, and the scanning delay was 8 s. Iopamidol was used as the contrast agent, with an injection volume of 90 mL and an injection rate of 5.0 mL/s. The scanning range covered from below the pubic symphysis to the supraclavicular area during a single breath-hold, and a scanning layer thickness of 0.8 mm. All patients signed informed consent forms and conducted iodine allergy experiments before the examination. CT scan images are stored in DICOM format on the hard disk.

CTA datasets were processed using 3D Slicer software (version 5.3.0). The arterial phase series providing optimal visualization of the DIEA was chosen for analysis. After adjustment of the display window and level to enhance vascular conspicuity, consecutive axial images covering the region from the DIEA origin to the umbilical level were exported as PNG images using the Screen Capture module. These PNG images were subsequently used for manual annotation and model development. Although no additional lossy compression was applied after PNG export, this display-based conversion does not preserve the complete quantitative Hounsfield-unit information or DICOM metadata contained in the original CTA data.

### 2.2. Vessel Annotation

**Figure 1:**
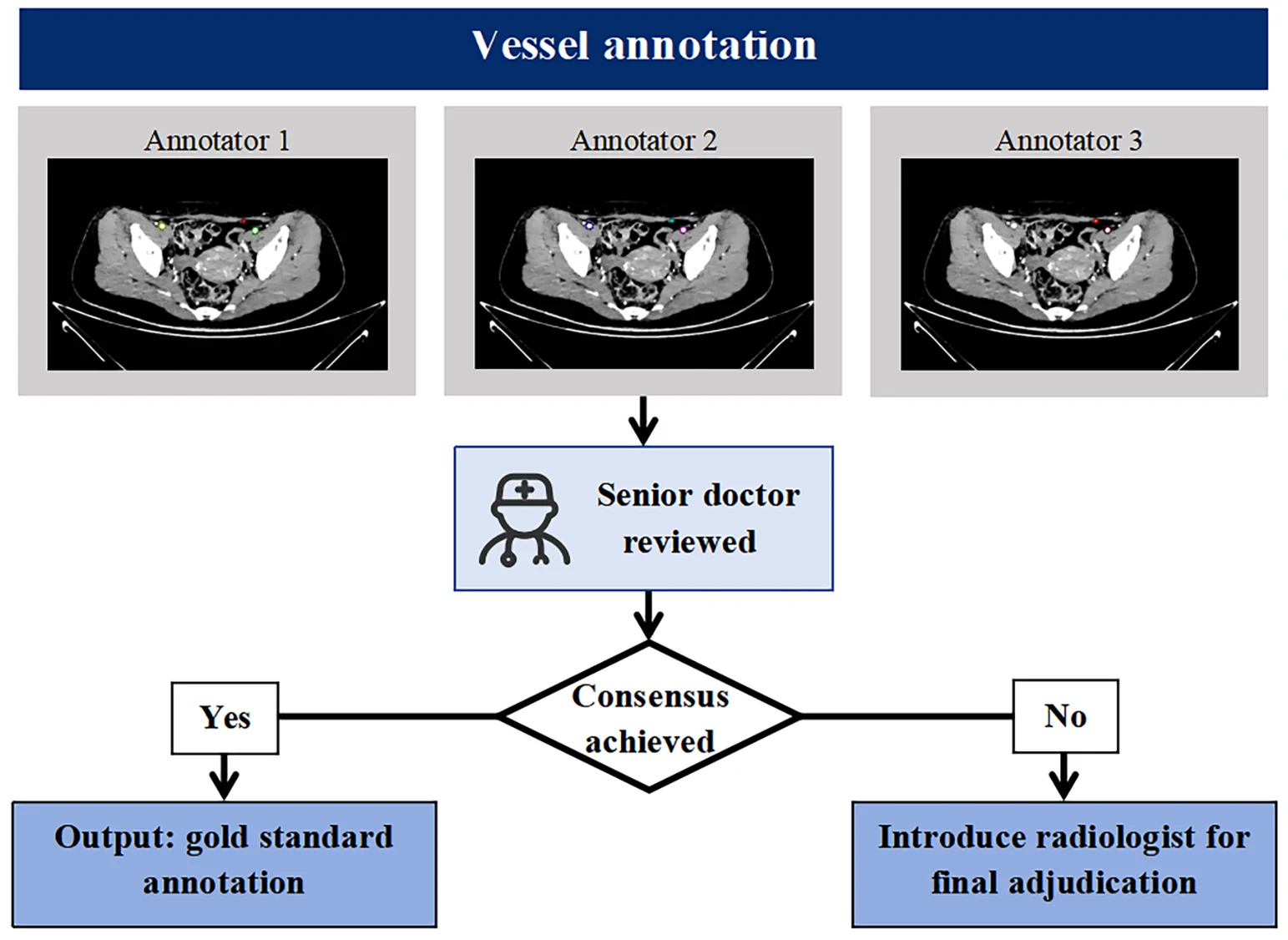
Flowchart of the collaborative quality control process for vessel segmentation and annotation.

A rigorous annotation protocol was employed using the LabelMe software (version 5.4.1), as depicted in Figure 1. Each image was independently segmented by three surgeons. All annotations were subsequently reviewed by a supervising physician. If a consensus was not reached among the initial annotations, a senior radiologist was consulted for final adjudication to establish a gold-standard segmentation and annotation result. The labeling encompassed the following vascular structures: the bilateral external iliac arteries (commencing from the bifurcation of the common iliac artery), the bilateral DIEA including their pubic branches (from their origin from the external iliac artery to the point where they pierce the anterior rectus sheath), and the bilateral deep iliac circumflex arteries (specifically at their junction with the external iliac artery). The labels of converging arteries were linked using a ’+’ symbol. Owing to insufficient image clarity and contrast, the vessel walls themselves were not annotated. After annotation, the 4446 CTA images were randomly assigned at the image level to training, validation, and test subsets using a 7:2:1 ratio.

### 2.3. Algorithm Development

**Figure 2:**
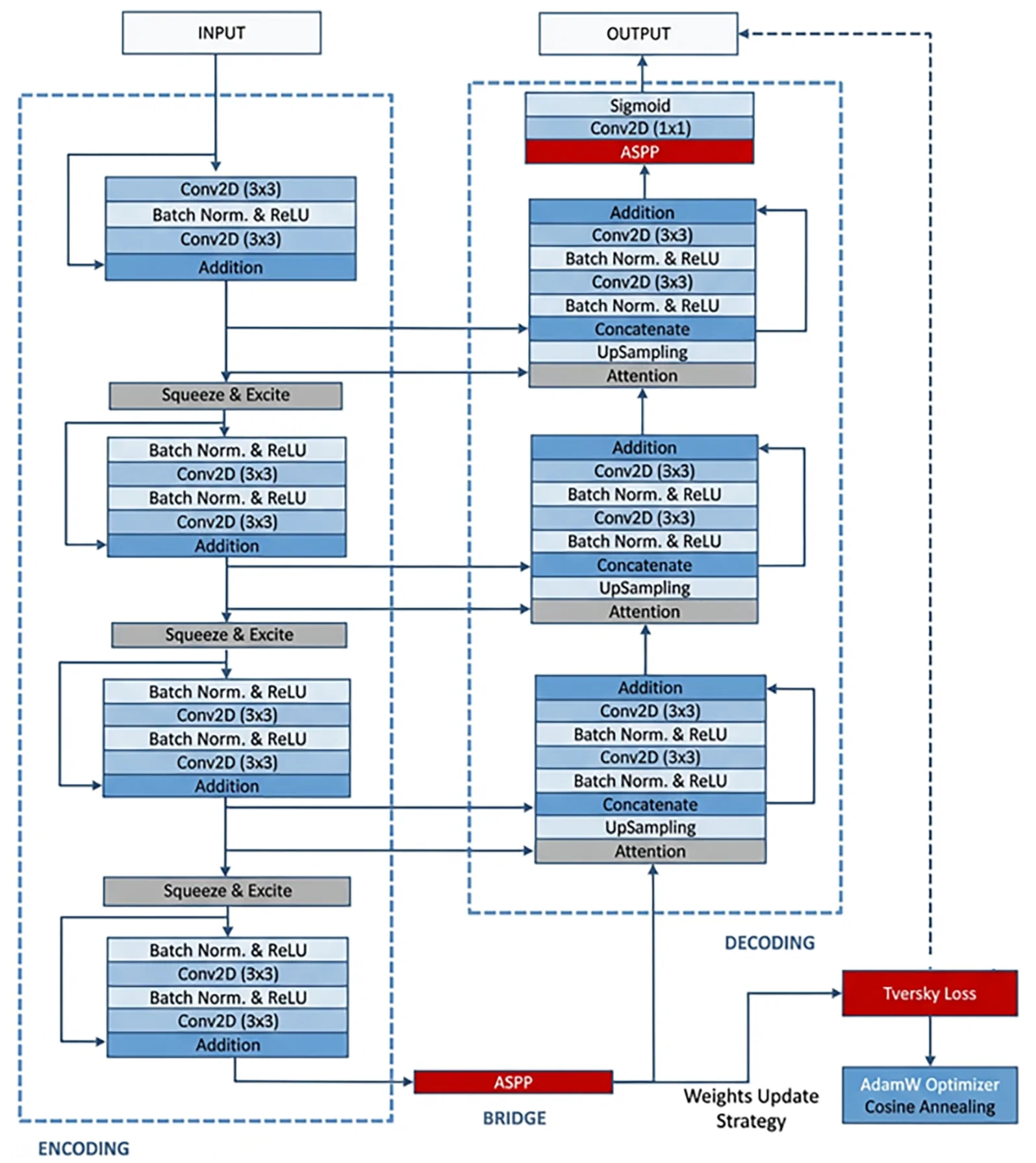
Architecture of the Improved ResU-Net++ Network with Enhanced Training Strategies.

The segmentation task was formulated as two-dimensional binary vascular foreground–background segmentation. All annotated vascular structures were merged into a single foreground class rather than treated as separate anatomical classes, consistent with the downstream objective of reconstructing the relevant vessels as a continuous vascular structure for preoperative visualization. Input images were resized to 256 × 256 pixels and represented as three-channel RGB images, while the network generated a single-channel foreground probability map of the same spatial size. Images were resized using bilinear interpolation and normalized by dividing pixel values by 255, whereas annotation masks were resized using nearest-neighbor interpolation. During evaluation, pixels with predicted probabilities greater than 0.5 were classified as foreground. To address the challenges of tiny vascular structures and the severe class imbalance (where vessel pixels are far outnumbered by background pixels) in CT vascular segmentation tasks, this paper proposes an improved ResU-Net++ framework enhanced with refined training strategies (Figure 2).

Network Architecture—The proposed network was based on ResU-Net++ and contained 14,482,564 trainable parameters. The input module consisted of two 3 × 3 convolutions and a residual branch, producing 32-channel features, with batch normalization (BN) and ReLU activation following the first convolution. The encoder contained three residual downsampling stages with output channels of 64, 128, and 256 at spatial resolutions of 128 × 128, 64 × 64, and 32 × 32, respectively. Each residual main branch used two BN–ReLU–3 × 3 convolution operations, with a stride of 2 in the first convolution, while the matching branch used a stride-2 3 × 3 convolution followed by BN. The decoder performed successive two-fold bilinear upsampling and concatenation with the corresponding encoder features, followed by residual convolution blocks producing 256, 128, and 64 channels. SE modules were applied before the three downsampling stages to the 32-, 64-, and 128-channel features. Each SE module used global average pooling followed by two fully connected layers with a reduction ratio of 16, ReLU activation in the intermediate layer, and Sigmoid activation for channel weighting. ASPP modules were placed at the end of the encoder and before the output stage. Each ASPP module contained three parallel 3 × 3 dilated convolution branches with dilation rates of 6, 12, and 18, followed by ReLU activation and BN; the concatenated features were fused using a 1 × 1 convolution. The bridge ASPP produced 512 channels, while the pre-output ASPP transformed 64-channel features to 32 channels. Three attention modules combined encoder and decoder features to generate single-channel spatial weighting maps, with max pooling applied to the encoder branch to match spatial dimensions. Finally, a 1 × 1 convolution followed by Sigmoid activation generated the foreground probability map.

Improved Loss Function—Conventional loss functions such as Cross Entropy or Dice Loss may provide insufficient penalty for false-negative predictions in highly imbalanced vascular segmentation, potentially resulting in missed or discontinuous vessel regions. To address this issue, we adopted Tversky Loss, which allows different weights to be assigned to false-negative and false-positive errors. Its mathematical definition is as follows:

(1) $$\mathrm{TL}\left(\alpha ,\beta \right)=1-\frac{\mathrm{TP}+1}{\mathrm{TP}+\alpha \mathrm{FN}+\beta \mathrm{FP}+1}.$$

where TP, FN, and FP denote true-positive, false-negative, and false-positive pixels, respectively. We set α=0.7, β=0.3, and the smoothing constant to 1, assigning greater weight to false negatives to reduce missed and discontinuous vessel regions.

Optimization and Training Strategy—We adopted AdamW instead of the standard Adam optimizer. By decoupling weight decay from gradient updates, AdamW provides more explicit regularization and may help reduce overfitting to noise in CTA images. In addition, a cosine annealing strategy was used to dynamically adjust the learning rate during training, allowing progressively smaller learning rates in later epochs and promoting more stable convergence.

Training Settings—The proposed model was trained for 50 epochs with a batch size of 8 and a random seed of 2026. AdamW was used with an initial learning rate of 0.001 and a weight decay of 0.0001, together with cosine annealing to a minimum learning rate of $1\times 10^{-6}$. No data augmentation or early stopping was applied, and the final-epoch weights were used for evaluation. Training used BF16 automatic mixed precision, while loss computation and model parameters were maintained in FP32. The implementation was conducted under WSL Ubuntu 22.04 using Python 3.10.12, PyTorch 2.9.1+cu128, and CUDA 12.8, with 8 GB of GPU memory.

Ablation Study—Six ablated variants were evaluated by individually removing SE, replacing ASPP with conventional convolutional blocks, removing attention, replacing Tversky loss with BCE+Dice loss, replacing AdamW with Adam, or replacing cosine annealing with a fixed learning rate of 0.001. All variants were trained under the same settings and evaluated on the same images.

### 2.4. Algorithm Evaluation

Model performance was evaluated using Sensitivity, Intersection over Union (IoU), Dice Similarity Coefficient, and Hausdorff Distance (HD). Pixel-wise Accuracy was additionally reported as a supplementary metric because of the marked foreground–background class imbalance. The proposed model was compared with U-Net, ResU-Net, and ResU-Net++ on the same evaluation dataset.

### 2.5. Statistical Analysis

Data were analyzed using SPSS 27.0. Continuous variables were assessed for normality and are presented as mean ± standard deviation (SD) or median (interquartile range, IQR), as appropriate, while categorical variables are presented as frequencies and percentages. The 95% confidence intervals (CIs) for segmentation metrics were estimated using nonparametric bootstrap resampling at the image level. Specifically, the 232 evaluation images were resampled with replacement 10,000 times using a random seed of 2026, and the 2.5th and 97.5th percentiles of the bootstrap distribution of the mean were used as the confidence limits. For the ablation comparisons, the same images were evaluated across model variants, with the image treated as the unit of analysis. Paired differences were assessed using two-sided Wilcoxon signed-rank tests, and Holm correction was applied across the 12 Dice and IoU comparisons. For the processing-time comparison, automated and manual processing times were paired at the image level and compared using a two-sided Wilcoxon signed-rank test. A two-sided p<0.05 was considered statistically significant.

## 3. Results

### 3.1. Dataset Characteristics

After applying the inclusion and exclusion criteria, the final study cohort comprised 31 patients (all female; mean age, 42.2 ± 10.8 years). From these patients, a total of 4446 abdominal CTA images were curated and annotated, forming the dedicated DIEA segmentation dataset. The images were randomly divided into training, validation, and test subsets at a 7:2:1 ratio. Because individual CTA images could contain multiple vascular structures, the distribution of specific labels did not strictly follow this ratio. The resulting label distributions are shown in Table 1.

### 3.2. Segmentation Performance

**Figure 3:**
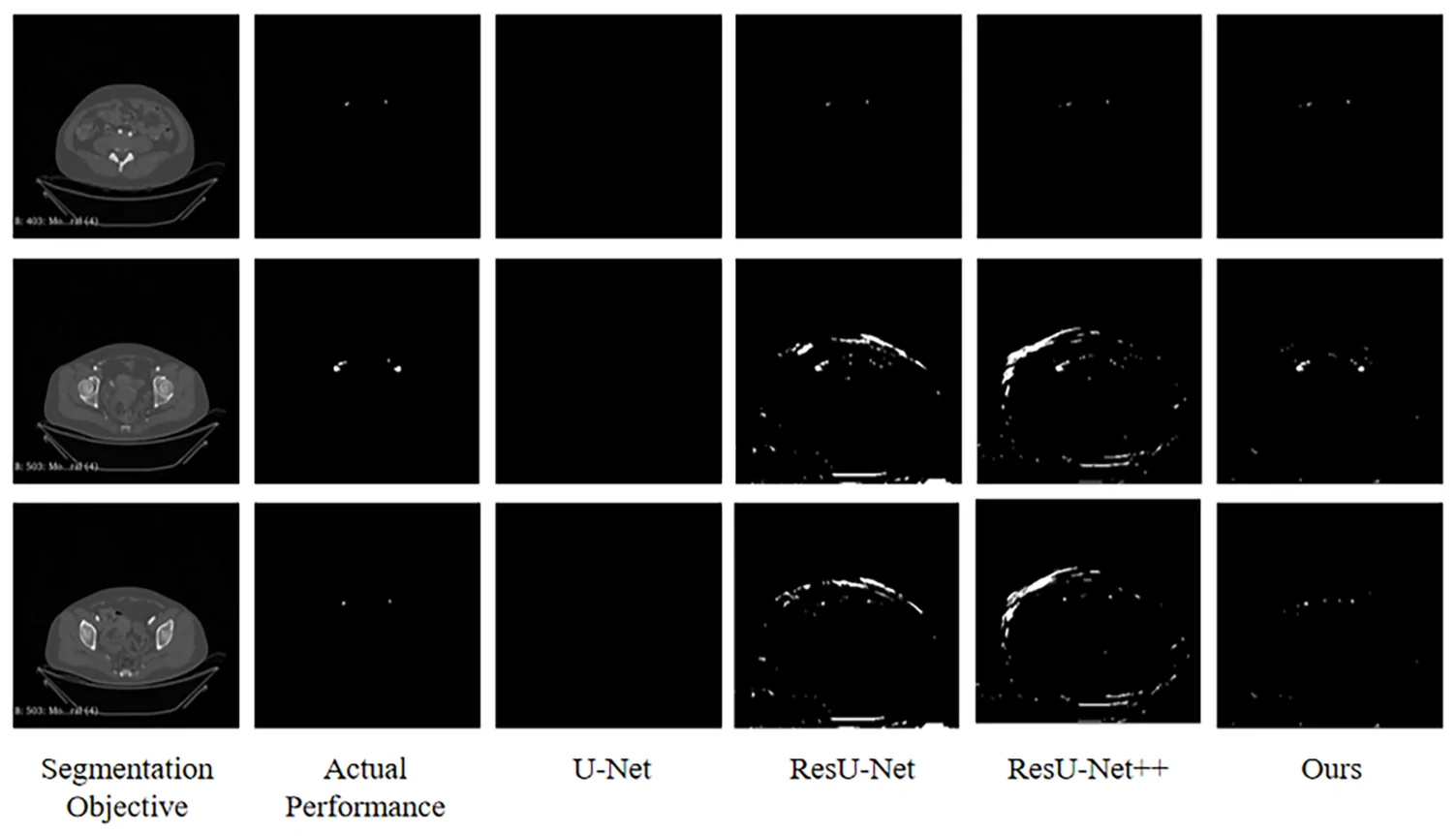
Segmentation Performance of Different Algorithms on Images.

The algorithm proposed in this study demonstrated strong performance in the segmentation of the DIEA and its associated vasculature (Figure 3). Compared with other segmentation networks (U-Net, ResU-Net, and ResU-Net++), the proposed model achieved superior segmentation performance across key metrics, including the Dice and HD (Table 2).

**Figure 4:**
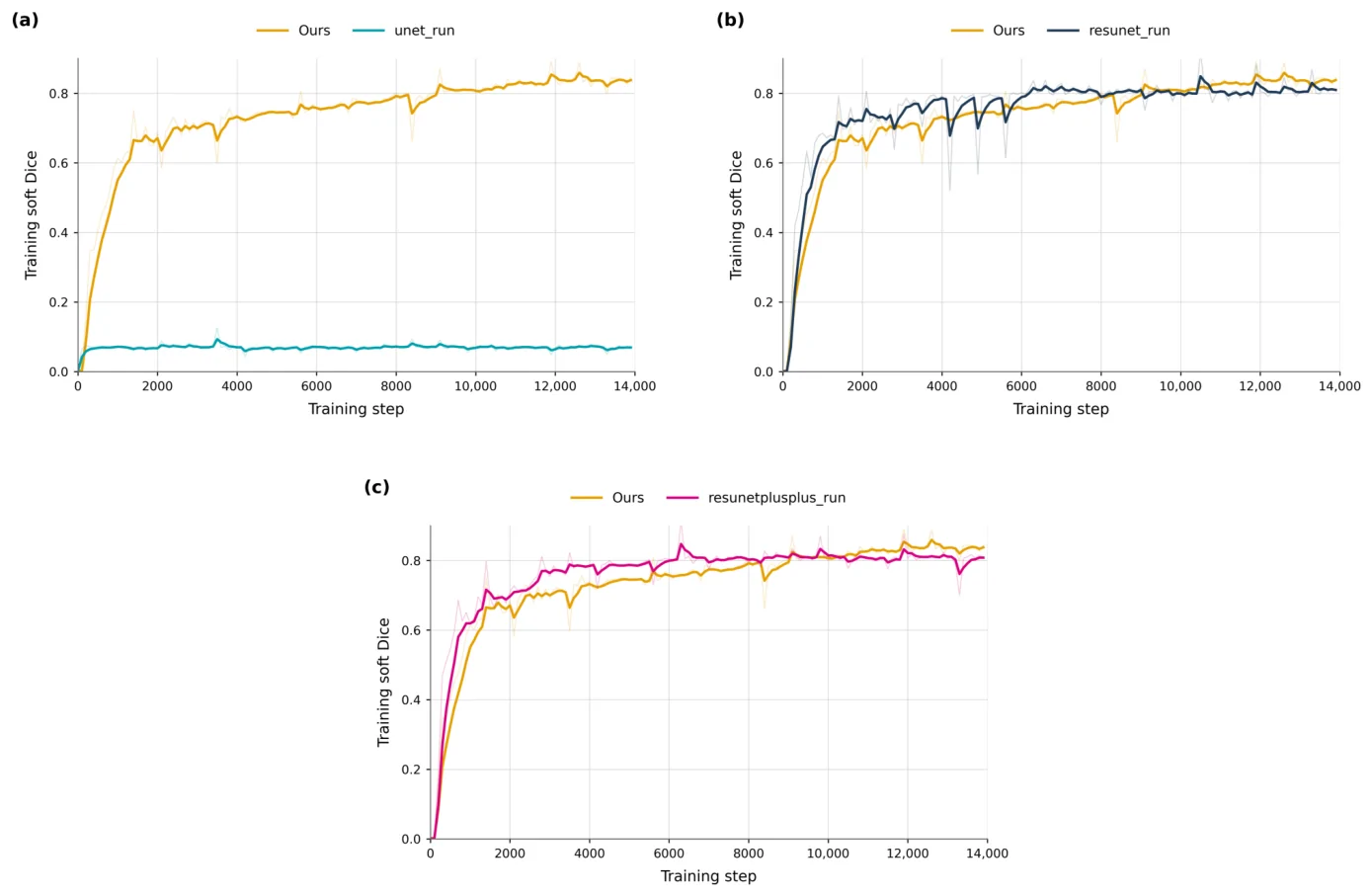
Comparison of training soft Dice scores across different model (**a**) Proposed model versus U-Net; (**b**) proposed model versus ResU-Net; (**c**) proposed model versus ResU-Net++. Darker curves represent smoothed values, whereas lighter curves represent unsmoothed values.

In a comparative analysis across different training steps among U-Net, ResU-Net, ResU-Net++, and the proposed enhanced ResU-Net++, the enhanced model consistently demonstrated higher IoU values during training (Figure 4).

### 3.3. Ablation Study

The full model achieved the highest Dice score of 0.6985. Ablating the SE modules, replacing ASPP with conventional convolutional blocks, removing the attention modules, substituting BCE+Dice loss for Tversky loss, replacing AdamW with Adam, and using a fixed learning rate all resulted in lower Dice scores. The corresponding reductions ranged from 0.73 to 7.19 percentage points. After Holm correction, the image-level Dice differences associated with SE, ASPP, attention, and cosine annealing were statistically significant, whereas those associated with Tversky loss and AdamW were not. Detailed quantitative results and adjusted p-values are presented in Table 3.

### 3.4. Model Processing Time

The complete automated processing pipeline, including data loading, network forward inference, and post-processing, required 0.0093±0.0015 s per image, compared with 19.85±5.75 s for manual segmentation. Using the image as the unit of analysis, a two-sided Wilcoxon signed-rank test showed that automated processing was significantly faster than manual segmentation (p<0.001). The mean reduction in processing time was 19.84 s per image (95% CI, 19.67–20.01 s).

### 3.5. Clinical Case Demonstration

We selected a representative abdominal CTA case to qualitatively demonstrate the clinical applicability of the proposed segmentation framework. The DIEA is characterized by a relatively small diameter, tortuous course, and close spatial relationship with surrounding muscles and soft tissues, making accurate delineation on conventional 2D CTA images challenging.

**Figure 5:**
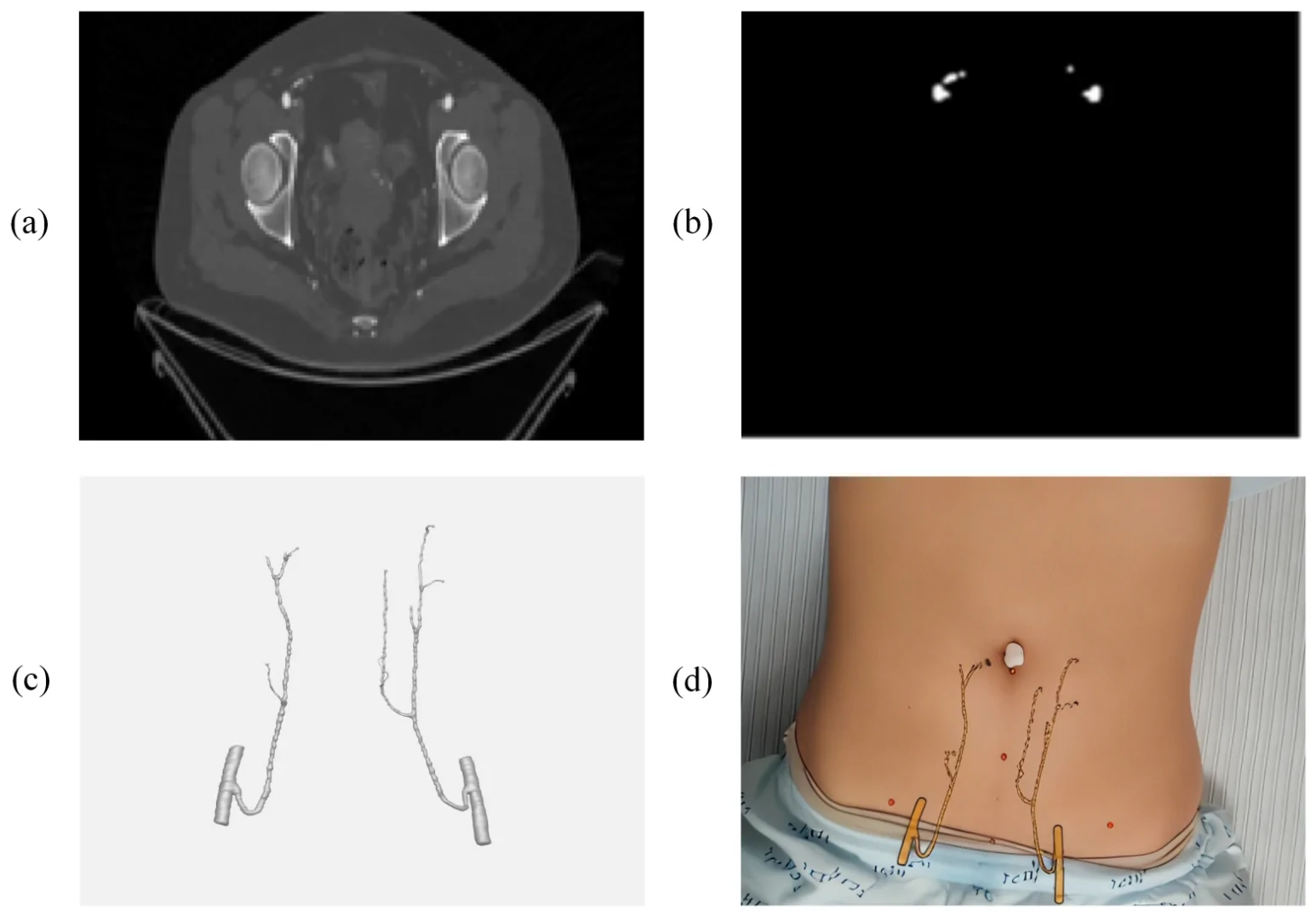
Clinical demonstration of automated segmentation from abdominal CTA images. (**a**) Original CTA image; (**b**) manual segmentation by experienced clinicians; (**c**) automated segmentation result generated by the proposed deep learning model; (**d**) three-dimensional reconstruction of the DIEA projected onto the patient’s abdominal surface.

As shown in Figure 5, the proposed model successfully identified and segmented the DIEA with good spatial continuity, and the resulting vessel trajectory showed close visual agreement with the expert manual annotation. In addition to the two-dimensional segmentation results, the automatically extracted vascular information was further used to generate a three-dimensional reconstruction of the DIEA. The reconstructed vessel was subsequently projected onto the patient’s abdominal surface for registration, providing an intuitive visualization of the vascular course and its spatial relationship with the abdominal wall. This visualization may assist surgeons in identifying the approximate location and course of the DIEA and in selecting potentially suitable perforators during preoperative planning. Compared with inspection of individual CTA slices alone, the three-dimensional representation provides a more intuitive overview of the vascular anatomy. This representative case therefore illustrates the potential of the proposed automated segmentation approach to serve as an intermediate step between CTA image analysis and clinically interpretable vascular visualization.

## 4. Discussion

Based on CTA vascular image segmentation, image analysis, and reporting is a labor-intensive process that can be challenging and requires specific expertise [17]. This study contributes a dedicated CTA image dataset for the DIEA to this field. This dataset comprises over 4000 high-quality images, each with pixel-level manual annotations performed by medical experts. The establishment of this standardized dataset provides a crucial foundation for the development and evaluation of automated segmentation algorithms specifically targeting this vessel.

To address the challenges of tiny vascular structures and the severe class imbalance in CT vascular segmentation tasks, this paper proposes an improved ResU-Net++ framework enhanced with refined training strategies. The framework utilizes ResU-Net++ as its backbone network, leveraging its powerful multi-scale feature extraction capabilities [18,19,20]. Building upon this foundation, we innovatively introduce Tversky Loss to mitigate vessel discontinuity issues, and combine the AdamW optimizer with a cosine annealing strategy to enhance the model’s generalization capability and convergence precision [21,22,23]. This model was trained on a dataset comprising more than 4000 images. The proposed model showed improved Dice, IoU, and Sensitivity relative to the evaluated baseline architectures within the present dataset. The performance improvement demonstrated by the algorithm proposed in this study for the task of DIEA segmentation stems from the systematic encoding of the specific clinical segmentation challenges and anatomical prior knowledge associated with this vessel into the design and training strategy of the deep learning model. First, the architectural enhancements directly address the core difficulties in identifying the DIEA in CTA images: its small diameter, low contrast against surrounding muscles and soft tissues, and tortuous course. To this end, we enhanced the ResU-Net++ backbone network and further optimized the training objective at the loss-function level, thereby improving the connectivity of the segmentation results. The SE module effectively suppresses background noise from the complex soft tissues of the abdominal wall and enhances vascular features through channel-wise recalibration. The ASPP module integrates local details with global context via multi-scale dilated convolutions, enabling the model to distinguish subtle vessels from noise and comprehend their three-dimensional trajectory. The Attention Gate mechanism utilizes deep semantic features to filter shallow ones, allowing the model to focus on vascular boundaries during reconstruction, thereby optimizing segmentation precision. To address the issues of missed detection and fragmentation caused by the extreme imbalance between foreground and background pixels, we adopted the Tversky loss function in place of the generic Dice loss. In our implementation, α and β were set to 0.7 and 0.3, respectively, assigning greater weight to false-negative errors to reduce missed vessel pixels and improve segmentation continuity. Regarding the training strategy, the AdamW optimizer was employed to more effectively constrain model complexity, preventing overfitting to the inherent noise in clinical CTA data and enhancing generalization capability. Combined with cosine annealing learning rate scheduling, this promotes stable convergence of the model to a superior performance plateau.

This study has several limitations. First, although 4446 CTA images provided substantial image-level information for model training, these images were derived from only 31 patients at a single center and were acquired using a single CT scanner and acquisition protocol. Thus, the independent clinical sample size and imaging diversity were limited, and no external validation was performed. These factors may limit the generalizability of the reported performance, and further validation using independent multicenter and multi-scanner datasets is warranted [24]. Second, during the data screening process, we excluded cases with poor image quality and suboptimal contrast opacification. While this ensured the quality of the training data, it also implies that the performance of the developed model in handling such complex cases remains unknown, and its efficacy in real-world applications across the full spectrum of patients requires further validation. Third, the model was developed using PNG images exported from the original DICOM data, which may result in loss of quantitative intensity information and DICOM metadata. This information loss was not quantitatively assessed. In addition, the dataset was partitioned at the image level using a single fixed split without cross-validation. As images from the same patient could be included in different subsets, within-patient similarity may have influenced the performance estimates. Although image-level 95% CI were reported, patient-level performance distributions and confidence intervals were not evaluated, limiting the assessment of between-patient variability. Finally, this study focused on the construction of a standardized database and the development of the algorithm itself. The seamless integration of the algorithm’s processing efficiency into existing clinical workflows is a key issue to be addressed in the next stage for clinical translation. Importantly, the present study did not evaluate the accuracy of surgical planning, operative time, surgical complications, flap outcomes, or other clinical endpoints. Therefore, the potential clinical benefits of automated vascular segmentation remain to be established in prospective clinical studies.

## 5. Conclusions

This study constructed a dedicated CTA dataset for DIEA segmentation and evaluated an automated segmentation model developed using this dataset. The findings demonstrate the technical feasibility of automated vascular segmentation within the present dataset. Further multicenter and prospective clinical studies are required to evaluate its generalizability and to determine whether automated segmentation can improve DIEP flap preoperative planning or clinical outcomes.

## Figures and Tables

**Table 1:** Distribution of Vascular Labels across the Training, Validation, and Test Subsets.

Label	Total	Training Set (%)	Validation Set (%)	Test Set (%)
EIA-R	2672	1972 (73.8)	443 (16.6)	257 (9.6)
EIA-L	2538	1826 (71.9)	432 (17.0)	280 (11.0)
DIEA-R	6027	4039 (67.0)	1396 (23.2)	592 (9.8)
DIEA-L	6080	4193 (69.0)	1366 (22.5)	521 (8.6)
EIA-R+DIEA-R	182	140 (76.9)	32 (17.6)	10 (5.5)
EIA-L+DIEA-L	173	141 (81.5)	27 (15.6)	5 (2.9)
EIA-R+DICA-R+DIEA-R	25	18 (72.0)	5 (20.0)	2 (8.0)
EIA-L+DICA-L+DIEA-L	20	19 (95.0)	1 (5.0)	0 (0)
EIA-R+DICA-R	35	21 (60.0)	14 (40.0)	0 (0)
EIA-L+DICA-L	36	22 (61.1)	13 (36.1)	1 (2.8)
DIEA-R(PB)	33	14 (42.4)	19 (57.6)	0 (0)
DIEA-L(PB)	11	11 (100.0)	0 (0)	0 (0)

Note: EIA = external iliac arteries; DIEA = deep inferior epigastric artery; DICA = deep iliac circumflex artery; PB = pubic branch.

**Table 2:** Comparative Evaluation of Vascular Segmentation Algorithms.

Model	Accuracy	Sensitivity	IoU	Dice	HD
U-Net	0.9996 (0.9996–0.9997)	0.0000 (0.0000–0.0000)	0.0000 (0.0000–0.0000)	0.0000 (0.0000–0.0000)	0.0000 (0.0000–0.0000)
ResU-Net	0.9998 (0.9998–0.9998)	0.6449 (0.6081–0.6809)	0.5459 (0.5142–0.5768)	0.6681 (0.6347–0.6997)	1.5734 (1.4962–1.6525)
ResU-Net++	0.9998 (0.9998–0.9998)	0.6558 (0.6202–0.6906)	0.5323 (0.5029–0.5601)	0.6615 (0.6306–0.6903)	1.6125 (1.5344–1.6895)
Ours	0.9998 (0.9998–0.9998)	0.7326 (0.6949–0.7686)	0.5556 (0.5266–0.5837)	0.6807 (0.6489–0.7105)	1.6207 (1.5503–1.6921)

Note: Values are presented as mean (95% CI). The 95% CIs were estimated using image-level nonparametric bootstrap resampling of the 232 evaluation images with replacement for 10,000 repetitions (random seed = 2026).

**Table 3:** Ablation Study of the Proposed Model.

Model Variant	Dice	IoU	Sensitivity	Precision	ΔDice (pp)	Adjusted *p*
Full model	0.6985	0.5643	0.7343	0.7204	–	–
Without SE	0.6792	0.5406	0.7199	0.6979	−1.94	$2.06\times 10^{-4}$
ASPP replaced	0.6622	0.5222	0.7461	0.6406	−3.63	$1.69\times 10^{-7}$
Without attention	0.6661	0.5252	0.7316	0.6579	−3.24	$4.87\times 10^{-5}$
BCE+Dice loss	0.6866	0.5573	0.6618	0.7793	−1.20	0.282
Adam	0.6913	0.5548	0.7478	0.6766	−0.73	0.088
Fixed learning rate	0.6266	0.4906	0.6334	0.7098	−7.19	$2.02\times 10^{-21}$

Note: ΔDice represents the change in Dice, in percentage points (pp), relative to the full model. Statistical comparisons were performed at the image level using two-sided paired Wilcoxon signed-rank tests. Holm correction was applied across the 12 Dice and IoU comparisons, and the adjusted *p*-values for Dice are reported in the table. A two-sided adjusted p<0.05 was considered statistically significant.

## Data Availability

The data that support the findings of this study are available from the corresponding author upon reasonable request. The data are not publicly available due to patient privacy and ethical restrictions.
